# Traditional Chinese medicine pharmacovigilance in signal detection: decision tree-based data classification

**DOI:** 10.1186/s12911-018-0599-5

**Published:** 2018-03-09

**Authors:** Jian-Xiang Wei, Jing Wang, Yun-Xia Zhu, Jun Sun, Hou-Ming Xu, Ming Li

**Affiliations:** 1 School of Internet of Things, Nanjing University of Posts and Telecommunications, Nanjing, 210003 China; 2 School of Computer Science and Technology, School of Software, Nanjing University of Posts and Telecommunications, Nanjing, 210023 China; 3 Jiangsu Center for ADR Monitoring, Nanjing, 210002 China

**Keywords:** Adverse drug reaction, Signal detection, Data classification, Decision tree

## Abstract

**Background:**

Traditional Chinese Medicine (TCM) is a style of traditional medicine informed by modern medicine but built on a foundation of more than 2500 years of Chinese medical practice. According to statistics, TCM accounts for approximately 14% of total adverse drug reaction (ADR) spontaneous reporting data in China. Because of the complexity of the components in TCM formula, which makes it essentially different from Western medicine, it is critical to determine whether ADR reports of TCM should be analyzed independently.

**Methods:**

Reports in the Chinese spontaneous reporting database between 2010 and 2011 were selected. The dataset was processed and divided into the total sample (all data) and the subsample (including TCM data only). Four different ADR signal detection methods-PRR, ROR, MHRA and IC- currently widely used in China, were applied for signal detection on the two samples. By comparison of experimental results, three of them—PRR, MHRA and IC—were chosen to do the experiment. We designed several indicators for performance evaluation such as *R* (recall ratio), *P* (precision ratio), and *D* (discrepancy ratio) based on the reference database and then constructed a decision tree for data classification based on such indicators.

**Results:**

For PRR: *R*_1_-*R*_2_ = 0.72%, *P*_1_-*P*_2_ = 0.16% and *D* = 0.92%; For MHRA: *R*_1_-*R*_2_ = 0.97%, *P*_1_-*P*_2_ = 0.20% and *D* = 1.18%; For IC: *R*_1_-*R*_2_ = 1.44%, *P*_2_-*P*_1_ = 4.06% and *D* = 4.72%. The threshold of *R*,*P*and *D*is set as 2%, 2% and 3% respectively. Based on the decision tree, the results are “separation” for PRR, MHRA and IC.

**Conclusions:**

In order to improve the efficiency and accuracy of signal detection, we suggest that TCM data should be separated from the total sample when conducting analyses.

## Background

The World Health Organization (WHO) defines adverse drug reactions (ADRs) as harmful and unintended reactions, which occur from the standard use of medicinal dosages for prophylaxis, diagnosis or treatment of diseases, or for the modification of physiological functions. ADRs include side effects, toxic effects, residual effects, idiosyncratic reactions, multiple infections arising from anti-infectives, drug dependence, and carcinogenic and mutagenic actions [1]. It is very difficult to identify the potential drug risks in clinical tests due to a range of issues, such as small sample size, limited observation time and scope. Subsequently, unintended adverse reactions may occur during medication use posing further threats to health and causing a financial burden on the patients. To strengthen monitoring of ADRs, China has established the ADR Monitoring Center and carried out appropriate endeavors since 1989. China established a national network system for ADR monitoring in 2003. By the end of 2016, the number of spontaneous reports submitted through the network has reached nearly 10,750,000 and has been drastically increasing at a rate of 1 million per year.

Currently, the methods used in signal detection in China mainly include Proportional Reporting Ratio (PRR), Reporting Odds Ratio (ROR), Medicines and Healthcare products Regulatory Agency (MHRA) and Information Component (IC) [2–9]. These methods calculate signal scores, i.e., the values for PRR, ROR and IC, to assess whether a drug is significantly associated with an adverse event. These calculations or algorithms, so-called the disproportionality analyses or measures, however, differ from one another in that the PRR, MHRA and ROR are frequentist (non-Bayesian), whereas the IC is Bayesian [10]. Many scholars of China have applied these methods on Chinese ADR data, but consistency in the detection results is poor [11–13]. So, no signal detection method that conforms to ADR data quality in China has been established. The co-existence of multiple detection methods causes challenge in signal detection. Moreover, the Chinese ADR database includes all drugs such as TCM, Western medicine, and biological products, but signal detection is not categorized by drug types. Owing to the large volume of data (about ten million), the variety of methods and the complexity of data processing, the China National Center for ADR Monitoring requires approximately two weeks to perform signal detection for all data, and the large amount of selected signals still need to be manually analyzed by experts. Thus, the efficiency of signal discovery is very low. Presently, there is no research on whether ADR data can be categorized by drug type before signal detection.

Combining TCM pharmacology with modern technologies, more drug types and preparations have entered Chinese market increasingly. The data system of the China Food and Drug Administration (CFDA) has shown 60,029 authorized TCMs as of October 17, 2013 [14]. Because of increased usage, the lack of knowledge about TCM and misuse, there is a surge in the amount of TCM ADR reports and the types of drugs involved. In the past few years, the CFDA has warned about the severe adverse reactions of Xiangdan, Shengmai, Xiyanping, Mailuoning, and Honghua (*Safflower*) injections, the safety issues of Lei Gong Teng (*Tripterygium wilfordii*) preparation, and the digestive reactions of the compound Qingdai pill. For example, Shengmai injection is a traditional Chinese medicine injection composed of Red Ginseng, *Ophiopogon japonicus* and Schisandra Chinensis. The adverse reactions of Shengmai injection in severe cases are as follows: body as a whole-general disorders accounted for about 53.2%, respiratory system disorders accounted for 20.7% and cardiovascular disorders accounted for about 11.4%. A total of 179 cases of Shengmai injection were reported, including anaphylactic shock (90) and severe anaphylactoid reaction (89), accounting for about 35.2% of all severe cases [15].

TCMs can be relatively crude preparations usually prepared as formula of numerous herbal and other natural-source ingredients, and as patent medicines i.e. manufactured, formulated products also containing numerous ingredients (sometimes they include conventional drug ingredients). TCMs have a botanical name, prescription name, trade name and pharmaceutical name. Usually, their names include Chinese names, Pin Yin names and Latin names. In China, generic drug names refer to China Approved Drug Names (CADN), which are the legal names of drugs formulated by the Pharmacopoeia Committee in accordance with the principle of CADN and submitted to the Ministry of public health for the record. All drugs composed of the same ingredients or the same formula have generic drug names are mandatory and binding. Generic drug names must be used on the label, instruction or package of listed drugs. So, each TCM in the spontaneous reporting data owns a unique generic drug name, named by Chinese characters (includes Pin Yin). ADR signal detection is based on generic drug names. In contrast with conventional medicines, TCMs are chemically rich complex mixtures comprising several hundreds of constituents, often more [16]. Owing to the inherent characteristics of TCM, the factors influencing its ADRs are more complicated than Western medicine. An understanding of the evaluation criteria on TCM ADR has always been vague. There is a lack of scientific, objective, and unbiased methods to evaluate TCM ADR [17, 18]. Some experts have pointed out that because of the special nature of TCM, its ADR data are quite different from those of Western medicine; thus, its ADR signal detection should be performed separately [16, 19]. The purpose of this paper is to solve the problem whether TCM should be separated for signal detection.

## Methods

### Data resource

A total of 1,823,144 ADR reports from 2010 and 2011 were obtained from the CFDA. Of these, 608,710 (33.4%) reports had one drug linked to multiple ADRs. The reports were further split into one drug to one ADR relationship. Reports with “unknown” drug name or ADRs were excluded. Following data processing, a total of 2,221,942 records were obtained. There are 317,417 records of TCM accounting for 14.29%; 1,874,904 records of “Western medicine” accounting for 84.38%; and 29,621 records of “biological products” accounting for 1.33%. The overall data were aggregated based on generic drug names of drugs and names of ADRs; as such, 139,281 drug-ADR pairs with their corresponding frequencies were obtained: this data set is Data1. This data set included 6174 drugs and 2458 ADRs.

In order to determine the effectiveness of classifying data for signal detection, a reference database of known ADRs needed to be established for comparison. The reference database was obtained from the CFDA and it was extracted from drug product labeling manually. The ADRs in the drug product labeling were collected primarily from pre-market clinical trials. In addition, side effects detected by the ADR monitoring system and verified by the experts would also be added to the drug product labeling during post-marketing. The drug name and ADR name of the reference database were mapped to the spontaneous reporting data. It includes 53,774 drug-ADR pairs and involves 2401 different drugs and 2460 different ADRs. The reference database will be noted as Data2.

Based on the two datasets, we make the further data processing as follows:

1. For Data1, because usually a minimum occurrence of 3 is accepted in signal detection, ADR data with an occurrence of less than 3 were removed from Data1. Further, for consistency in the reference database, drugs that were present in Data1 but absent in Data2 were removed from Data1. This resulted in a dataset with 39,782 records, involving 1692 different drugs and 877 ADRs. This dataset is referred to as the total sample.
2. TCM data were extracted from the total sample, generating a TCM dataset. The dataset included 4697 records, involving 326 drugs and 283 ADRs. This dataset is referred to as the subsample, and it is a proper subset of the total sample.
3. Fields in the two datasets include “type of drug”, “generic drug name”, “name of ADR”, “occurrence frequency”, “known or not”, etc. The Data2 is used to annotate the field “known or not” in the total sample and the subsample: if the drug-ADR pair that appeared in the reference database, it was annotated as “1,” otherwise it was annotated as “0.”

### Selection of signal detection methods

Currently, ADR signal detection methods in China utilize such four mainstream methods as: PRR, ROR, MHRA, and IC, and the calculations of measures of disproportionality are primarily based upon a two-by-two contingency table (Table 1) [20].

**Table 1:** Two-by-two contingency table

	Target ADRs	Other ADRs	Total
Target drugs	*a*	*b*	*a + b*
Other drugs	*c*	*d*	*c + d*
Total	*a + c*	*b + d*	*a + b + c + d*

The four methods are applied to the subsample respectively. The signal detection of the subsample was done at the TCM product level. The results of positive signals are listed in Table 2.

**Table 2:** The number of positive signals in the two samples, detected by the four methods

Signal detection methods	The number of positive signals in the total sample	The number of positive signals in the subsample
PRR+	18,780	2209
ROR+	18,568	2145
MHRA+	15,952	1820
IC+	7196	660

As shown in Table 2, the numbers of positive signals generated by PRR, ROR, MHRA and IC showed a decreasing relationship. Using “1” to indicate positive signal and “0” to indicate non-positive signal, the correlation coefficients of the detection results of the four methods are shown in Table 3 and Table 4.

**Table 3:** Correlation coefficients of four methods in the total sample

	PRR	ROR	MHRA	IC
PRR	1			
ROR	0.9887	1		
MHRA	0.8670	0.8613	1	
IC	0.5009	0.5055	0.5069	1

**Table 4:** Correlation coefficients of four methods in the subsample

	PRR	ROR	MHRA	IC
PRR	1			
ROR	0.9721	1		
MHRA	0.8441	0.8219	1	
IC	0.4205	0.4275	0.4241	1

We knew from Tables 3 and 4 that the correlation coefficient of PRR and ROR was close to 1(0.9887 and 0.9721), that is, the outcomes of the two methods are basically identical. At the same time, the two approaches are based on the theory of disproportionality. Therefore, it’s necessary to use both PRR and POR. We chose PRR, MHRA, and IC as the three methods for signal detection in this study. All the methods of signal detection were implemented with Visual FoxPro database software 6.0.

### Decision-making process

**Fig. 1:**
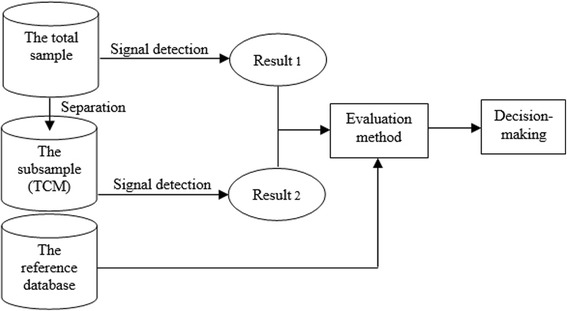
Decision-making process flowchart

To judge the superiority and inferiority of the signal detection outcomes in the two samples before and after data separation, the following issues needed to be addressed: How to evaluate the difference of results of signal detection methods applied to the two samples before and after data separation? What indicators were needed to be set for discrimination? What was the decision- making process? Flowchart on decision-making process is presented as follows (Fig. 1).

Detecting signals in the total sample and the subsample respectively. Then, two results were obtained. Some indicators of evaluation method were constructed and used to evaluate the superiority and inferiority of the two results. The decision-making was conducted based on the reference database and the evaluation results.

### Fourfold table design based on the reference database

The reference database provided an objective evidence for classification decisions. Signal detection of the two samples was performed and compared with the reference database, which could be structured into a fourfold table as follows (Table 5).

**Table 5:** Two-by-two contingency table of the two samples

	The subsample +	The subsample -
The total sample +	*a*(*a*_1_, *a*_0_)	*b*(*b*_1_, *b*_0_)
The total sample -	*c*(*c*_1_, *c*_0_)	*d*(*d*_1_, *d*_0_)

In Table 5, *a* represents the number of ADR pairs with positive signals in both the total sample and the subsample, *a*_1_ represents the number of ADR pairs that are present in the total sample, the subsample, and the reference database, *a*_0_ represents the number of ADR pairs that are present in the total sample and the subsample but are absent in the reference database, and *a* = *a*_1_ + *a*_0_. The other symbols such as *b*(*b*_1_, *b*_0_), *c*(*c*_1_, *c*_0_) and *d*(*d*_1_, *d*_0_) are analogous to *a*(*a*_1_, *a*_0_). The ADR pairs in *b*_1_ and *c*_1_ are distinct signals between two samples, and thus can be used as a basis for classification decision.

### Evaluation method

1. Indicator *R - Recall ratio*

The recall ratio, a measure of the coverage of known signals, represents the ratio of the signals detected from the reference database. Using recall ratio *R*_1_ to describe the ability to detect known signals in the total sample, as represented by formula (1):

1 $$ {R}_1=\frac{a_1+{b}_1}{a_1+{b}_1+{c}_1+{d}_1} $$

From Table 5, we can see that *a*_1_ + *b*_1_ + *c*_1_ + *d*_1_ is the total number of known signals and *a*_1_ + *b*_1_ is the number of known signals detected in the total sample. Similarly, using recall ratio *R*_2_ to describe the ability to detect known signals in the subsample, as represented by formula (2):

2 $$ {R}_2=\frac{a_1+{c}_1}{a_1+{b}_1+{c}_1+{d}_1} $$

By comparing the recall ratios between the two samples, the differences in the ability to detect known signals can be distinguished, which also reflect the differences in signal detection sensitivities between these two samples based on the reference database. Therefore, the recall ratio is a key indicator that should be used as the primary basis for classification decisions.

2) Indicator *P* - *Precision ratio*

The drawback of formula (1) and (2) lies in the fact that when *a*_1_ is much greater than *b*_1_ and *c*_1,_ there is no significant difference between *b*_1_ and *c*_1_ even though they have markedly distinct values. Therefore, if the difference between *R*_1_ and *R*_2_ is slight, it is necessary to define the precision ratio. The precision ratio, a measure of the accuracy of detecting known signals, is the proportion of the known signals detected based on a certain sample. We use precision ratio *P*_1_ to describe the ability to detect known signals in the total sample, which is represented by formula (3).

3 $$ {P}_1=\frac{a_1+{b}_1}{a+b} $$

From Table 5, we can see that *a* + *b* is the total number of signals detected in the total sample and *a*_1_ + *b*_1_ is the number of known signal detected in the total sample. Similarly, using precision ratio *P*_2_ to describe the ability to detect known signals in the subsample, as represented by formula (4).

4 $$ {P}_2=\frac{a_1+{c}_1}{a+c} $$

3) Indicator *D - discrepancy ratio*

Discrepancy ratio represents the diversity measurement of the detection outcomes in the two samples, represented by formula (5).

5 $$ D=\mid \frac{b_1-{c}_1}{b+c}\mid $$

In formula (5), *b* + *c* represent the total number of different detection outcomes in the two samples.

### Decision tree

Using the indicators of *R*, *P* and *D* in the total sample and the subsample to decide whether to classify TCM data, the process is as follows.

1. The primary basis is to consider the coverage percentage of known signals based on the detected outcomes in the two samples. Specifically, the indicator *R* of the two samples is considered (See formula 1 and 2). Sample with high recall ratio is chosen. If *R*_1_ − *R*_2_ > *R*_*t*_, it is recommended to not perform classified detection of TCM data; if *R*_2_ − *R*_1_ > *R*_*t*_, it is recommended to perform classified detection of TCM data; if the recall ratio difference is slight, further decision is needed (*R*_*t*_ represents the threshold of *R*).
2. When there is little difference in recall ratios of the two samples, the indicator *P* of the signal detection needs to be considered, which is the precision ratio of the two samples (See formula 3 and 4). If *P*_1_ − *P*_2_ > *P*_*t*_, it is recommended to not perform classified detection of TCM data; if *P*_2_ − *P*_1_ > *P*_*t*_, it is recommended to perform classified detection of TCM data; if the precision ratio difference is slight, further decision is needed (*P*_*t*_ represents the threshold of *P*).
3. When there is little difference in both recall ratio and precision ratio of the two samples, then indicator *D* is compared. If the discrepancy ratio exceeds the prescribed threshold (*D* > *D*_*t*_), the decision outcome is “non-separation”, otherwise is “separation” (*D*_*t*_ represents the threshold of *D*).

**Fig. 2:**
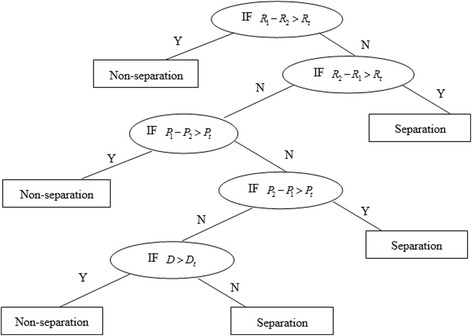
Decision tree for determining whether to classify data. Note: *R*_1_ and *R*_2_ represent the recall ratios of the total sample and the subsample. *P*_1_ and *P*_2_ are the precision ratios of two samples. *D* is the discrepancy ratio of two samples. *R*_*t*_, *P*_*t*_ and *D*_*t*_ represent the threshold of each indicator respectively

Based on the above analysis, the decision tree for determining whether to perform classified detection of data is constructed as Fig. 2.

## Results

The statistical results of the two samples are shown in Table 6 and Table 7.

**Table 6:** Statistical results of the two samples

Type	The total sample	The subsample
ADR reports	1,972,008	199,115
Drugs involved	1692	326
ADRs involved	877	283
Drug-ADR pairs (known drug-ADR pairs)	39,782 (13,555)	4697 (830)
Serious ADR reports	88,083	10,007

**Table 7:** Top ten ADRs with highest frequency in two samples

Adverse reaction	Frequency of ADRs in the total sample (proportion)	ADR	Frequency of ADRs in the subsample (proportion)
Rash	281,399 (14.27%)	Rash	32,161 (16.15%)
Nausea	226,368 (11.48%)	Pruritus	22,736 (11.42%)
Pruritus	173,111 (8.78%)	Nausea	14,339 (7.20%)
Vomiting	141,098 (7.16%)	Dizziness	9577 (4.81%)
Dizziness	83,278 (4.22%)	Shivering	9206 (4.62%)
Headache	56,507 (2.87%)	Palpitation	8728 (4.38%)
Abdominal pain	52,291 (2.65%)	Vomiting	8661 (4.35%)
Diarrhea	49,839 (2.53%)	Anaphylactoid reaction	7182 (3.61%)
Anaphylactoid reaction	49,261 (2.50%)	Chest tightness	6251 (3.14%)
Shivering	39,066 (1.98%)	Fever	5847 (2.94%)
Total	1,152,218 (58.44%)		124,688 (62.62%)

The PRR, MHRA, and IC methods are used to perform experiments with the total sample and the subsample and the results are shown in Table 8.

**Table 8:** Experiment results through the three detection methods

Detection method		The subsample +	The subsample -
PRR	The total sample +	*a* = 1888 (*a*_1_ = 561, *a*_0_ = 1327)	*b* = 330 (*b*_1_ = 40,*b*_0_ = 290)
	The total sample -	*c* = 321 (*c*_1_ = 34, *c*_0_ = 287)	*d* = 2158 (*d*_1_ = 195, *d*_0_ = 1963)
MHRA	The total sample +	*a* = 1488 (*a*_1_ = 456, *a*_0_ = 1032)	*b* = 348 (*b*_1_ = 56, *b*_0_ = 292)
	The total sample -	*c* = 332 (*c*_1_ = 48, *c*_0_ = 284)	*d* = 2529 (*d*_1_ = 270, *d*_0_ = 2259)
IC	The total sample +	*a* = 579 (*a*_1_ = 274, *a*_0_ = 305)	*b* = 173 (*b*_1_ = 43, *b*_0_ = 130)
	The total sample -	*c* = 81 (*c*_1_ = 31, *c*_0_ = 50)	*d* = 3864 (*d*_1_ = 482, *d*_0_ = 3382)

A decision-making table (Table 9) is established using data from Table 8 and the classification decision tree. The thresholds of three indicators are set based on the mean of the differences of *R*, *P* and *D* across three signal detection methods. For *R*, the difference of PRR, MHRA and IC is 0.72%, 0.97% and 1.44%, so the mean of three differences about equal to 1.04%. Thus, the threshold of *R* is set to 2%, an integer slightly larger than the mean. Similarly, for *P*, the mean of the differences is (0.16% + 0.20% + 4.06%)/3 ≈ 1.47%, so the threshold of *P* is set to 2%. For *D*, the mean of the differences is (0.92% + 1.18% + 4.72%) /3 ≈ 2.73%, so the threshold of *D* is set to 3%. In brief, we set *R*_*t*_ to 2%, *P*_*t*_ to 2% and *D*_*t*_ to 3% in the following decision process. Based on the decision tree (see Fig. 2), the decision-making process is as follows:

1. PRR: because *R*_1_-*R*_2_ = 0.72% < 2%, *P*_1_-*P*_2_ = 0.16% < 2% and *D* = 0.92% < 3%, the conclusion is “Separation”.
2. MHRA: because *R*_1_-*R*_2_ = 0.97% < 2%, *P*_1_-*P*_2_ = 0.20% < 2% and *D* = 1.18% < 3%, the conclusion is “Separation”.
3. IC: because *R*_1_-*R*_2_ = 1.44% < 2%, *P*_2_-*P*_1_ = 4.06% > 2%, the conclusion is “Separation”.

**Table 9:** Decision-making table

Detection method	Indicators	The total sample	The subsample	Difference
PRR	Recall ratio (%)	*R*_1_ = 72.41%	*R*_2_ = 71.69%	0.72%
	Precision ratio (%)	*P*_1_ = 27.10%	*P*_2_ = 26.94%	0.16%
	Discrepancy ratio (%)	*D* = 0.92%		
MHRA	Recall ratio (%)	*R*_1_ = 61.69%	*R*_2_ = 60.72%	0.97%
	Precision ratio (%)	*P*_1_ = 27.89%	*P*_2_ = 27.69%	0.20%
	Discrepancy ratio (%)	*D* = 1.18%		
IC	Recall ratio (%)	*R*_1_ = 38.19%	*R*_2_ = 36.75%	1.44%
	Precision ratio (%)	*P*_1_ = 42.15%	*P*_2_ = 46.21%	−4.06%
	Discrepancy ratio (%)	*D* = 4.72%		

Thus, the conclusion of “Separation” is reached by using all three methods.

## Discussion

### Comparative analysis of two samples

The statistical results of two samples are shown in Table 6.

The total sample includes 1,972,008 ADR reports, involving 1692 drugs, 39,782 drug-ADR pairs, and 877 ADRs. The average number of ADRs is 1165.49 for each drug and 49.57 for each drug-ADR pair, whereas each ADR has an average frequency of 2248.58.

The subsample is a collection of all reports of TCM removed from the total sample. It contains 199,115 ADR reports of TCM, accounting for 10.1% of the total sample, in which 326 drugs, 4697 drug-ADR pairs, and 283 ADRs are involved. The average number of ADRs is 610.78 for each drug and 42.39 for each drug-ADR pair, whereas each ADR has an average frequency of 703.59. Compared with the total sample, the average frequency of the subsample is respectively reduced by 554.7, 7.18 and 1545.

Compared with the reference database, the total sample contains 13,555 known drug-ADR pairs, and the proportion is 34.07%; the subsample contains 830 known drug-ADR pairs, and the proportion is 17.67%, 16.4% less than that in the total sample. Specifically, known drug-ADR pairs in the TCM database are far less than those in the total sample.

The total sample contains 88,083 serious reports, accounting for 4.47%. The subsample contains 10,007 serious reports, accounting for 5.03%. Therefore, the subsample has relatively higher serious report proportion than the total sample.

Table 7 shows the top ten ADRs with highest frequency in two samples. For each sample, the sum of their frequency is close to 60%. Seven of the top ten adverse reactions are the same between the two samples. The obvious difference between the two samples is that “headache” (2.87%), “abdominal pain” (2.65%) and “diarrhea” (2.53%) in the total sample, and “palpitation” (4.38%), “chest tightness” (3.14%) and “fever” (2.94%) in the subsample. The top three adverse reactions in both samples are “rash” (14.27% vs 16.15%), “nausea” (11.48% vs 7.20%) and “pruritus” (8.78% vs 11.42%), but the proportion of “nausea” is 4.28% higher in the total sample than that in the subsample, while “pruritus” is 2.64% lower than that in the subsample.

### Discussion of methods

This paper presents a decision method by comparing signal detection results before and after separating TCM data from the total sample. The method employs two important formulas of information retrieval theory: recall ratio and precision ratio. Recall ratio is an indicator of success which represents the proportion of known ADRs retrieved from all known TCM ADRs. Precision ratio refers to the proportion of known ADR in all retrieved signals, and is an indicator of signal-noise ratio. In the design of decision-making tree, we first consider the recall ratio, which is about whether more known signals can be detected with independent signal detection in TCM sample. If there is no difference in recall ratio, further consideration should be given to the precision ratio. In addition, the discrepancy ratio is added for decision-making when the above two indicators cannot work well. The decision method is based on the reference database and the proportion of known ADRs of TCM in it is very low (17.67%), this could result in uncertainty of the decision outcome. At the same time, according to the previous data analysis, there have been some differences in the two samples. Thus, according to the TCM data characteristics, the decision result is biased towards “separation” when the corresponding values of the three indicators are relatively close before and after data separation.

### Discussion of results

Table 8 shows the experiment results with the three detection methods. For PRR, the total number of signals detected in the total sample is 2209, the subsample is 2218, and the number of common signals is 1888. For MHRA, the number of signals detected in the total sample is 1820, the subsample is 1836, and the number of common signals is 1488, which is 389, 382, and 400 less than that of the PRR method. For IC, the total number of signals detected in the total sample is 660, the subsample is 752, and the number of common signals is 579. Obviously, among the three methods, IC detects the lowest number of signals.

Table 9 gives the values of all indicators of the three methods. Since the PRR method detects the most signals, it contains the largest number of known ADR, resulting in the highest recall ratio of 72.41%. The recall ratios of MHRA and IC are 61.69% and 38.19% respectively. But the precision ratio is opposite. Although the total amount of signals detected by IC is the smallest, its precision ratio is the highest, 42.15%, while the precision ratios of PRR and MHRA are 27.10% and 27.89% respectively.

For PRR and MHRA, decision-making paths are consistent and the results are all “Separation” because all the values of three indicators fall within the according thresholds. For IC, however, decision-making path is deferent from PRR and MHRA. The recall ratio of the two samples is very similar, but the subsample has a significantly high precision ratio than the total sample. So, the conclusion is “Separation” for IC.

The dataset of this research is fixed, that is to say, the total sample and the subsample are fixed, so the values of three indicators calculated based on two samples are constant, and they will not change with the different threshold. Thus, it is impossible to determine the optimum threshold in traditional ways such as Precision Recall curve, Receiver Operating Characteristic Curve or Mean Average Precision. In this paper, the choice of the threshold is mainly based on the mean value of the difference between the same indicator across all methods before and after data separation. If the ADR data of more years can be obtained, we can group data by increasing year by year to observe change trends of the difference value of the three indicators. It will help to determine the thresholds more accurately. This will be the further work we need to do.

### Limitations

Our research has a few limitations. First, the low quality of the ADR report data and the co-existence of multiple signal detection methods may bring uncertainty to the results. Second, this study is based on the data provided by the CFDA during 2010–2011. The limited amount of data (approximately 2.2 million) may not be representative of the total data of the CFDA (approximately 10 million). Third, another limitation of the study is the use of only the ADRs extracted from drug product labeling manually. In China, there is a requirement that all ADRs observed in clinical studies should be submitted as spontaneous reports and those verified by the experts should be added to the drug product labeling. But the reference database might not include the kinds of signals which are novel and unexpected associations that appear once a drug is used by a broader group of individuals.

## Conclusion

Whether TCM should be independent signal detection or not has become an important issue of pharmacovigilance in China. In order to address this problem, we propose a method based on decision tree in this study. We first analyze the results of applying four mainstream ADR signal detection methods that have been used widely in China and select three of these four methods. We set three indicators used for performance evaluation such as *R*, *P* and *D* based on the reference database, and then construct a decision tree for data classification. Finally, we conduct experiment on the total sample and the subsample (TCM). Experiment results show that the conclusions are all “Separation” for PRR, MHRA and IC. Thus, we suggest that TCM data should be separated from the total data when conducting analyses.

## Abbreviations

ADR: Adverse drug reaction
CADN: China approved drug names
CFDA: China food and drug administration
IC: Information component
MHRA: Medicines and healthcare products regulatory agency
PRR: Proportional reporting ratio
ROR: Reporting odds ratio
TCM: Traditional Chinese medicine
